# Clinical Effects of Activated Charcoal Unavailability on Treatment Outcomes for Oral Drug Poisoned Patients

**DOI:** 10.1155/2018/4642127

**Published:** 2018-10-03

**Authors:** Sohyun Park, Hui Jai Lee, Jonghwan Shin, Kyoung Min You, Se Jong Lee, Euigi Jung

**Affiliations:** ^1^Department of Emergency Medicine, Seoul Metropolitan Government-Seoul National University Boramae Medical Center, Seoul 07061, Republic of Korea; ^2^Department of Emergency Medicine, Sejong General Hospital, Bucheon, Gyeonggi-do 14754, Republic of Korea; ^3^Department of Emergency Medicine, Central Veterans Hospital, Seoul 05368, Republic of Korea

## Abstract

**Background:**

Activated charcoal is the most frequently and widely used oral decontaminating agent in emergency departments (EDs). However, there is some debate about its clinical benefits and risks. In Korea, activated charcoal with sorbitol was unavailable as of the mid-2015, and our hospital had been unable to use it from September 2015. This study examined the differences of clinical features and outcomes of patients during the periods charcoal was and was not available.

**Methods:**

We retrospectively reviewed the electronic medical records of patients who had visited an urban tertiary academic ED for oral drug poisoning between January 2013 and January 2017.

**Results:**

For the charcoal-available period, 413 patients were identified and for the charcoal-unavailable period, 221. Activated charcoal was used in the treatment of 141 patients (34%) during the available period. The mortality rates during the available and unavailable periods were 1.9 and 0.9%, respectively (*p* = 0.507). There was also no interperiod difference in the development of aspiration pneumonia (9.9 versus 9.5%,* p *= 0.864), the endotracheal intubation rate (8.4 versus 7.2%,* p *= 0.586), and vasopressor use (5.3 versus 5.0%,* p *= 0.85). Intensive care unit (ICU) admission was higher in the unavailable period (5.8 versus 13.6%,* p* = 0.001). ICU days were lower in the unavailable period (10 [4.5-19] versus 4 [3-9],* p* = 0.01). Hospital admission (43.3 versus 29.9%,* p *= 0.001) was lower in the unavailable period.

**Conclusions:**

In this single center study, there appeared to be no difference in mortality, intubation rates, or vasopressor use between the charcoal-available and charcoal-unavailable periods.

## 1. Introduction

Activated charcoal is the gastrointestinal (GI) decontaminating agent that had been regarded as an essential first-line therapy for acute-poisoning patients. However, few studies have shown clinical improvement of poisoned patients who had been treated using activated charcoal. Indications of activated charcoal are decreasing. The indications and use of activated charcoal as a decontamination procedure actually have been declining over the years [1–3]. American poison centers reported a sharp drop between 1995 and 2016, from 7.7% of all exposures to 1.9%, respectively [4]. The most recent guidelines emphasize that activated charcoal should not be used routinely [5]. However, there are as yet no specific and detailed guidelines on the use of activated charcoal. Indeed, there are significant variations in the use of activated charcoal among clinicians [6, 7].

In Korea, premixed activated charcoal with sorbitol had been the only available form of activated charcoal [8]. Due to importation issue, its use was discontinued from 2015. As a result, activated charcoal with sorbitol has been unavailable at our hospital from September of that year. In the present study, we evaluated the clinical outcome differences between the charcoal-available and charcoal-unavailable periods.

## 2. Materials and Methods

### 2.1. Study Design and Setting

This study was conducted in an urban academic teaching hospital with an annual emergency department (ED) census of 58000. A retrospective chart review was conducted for the period from January 2013 to January 2017. Patients whose ICD-10-based ED diagnosis was poison-related were selected from electronic medical records. Those who had visited the ED with oral drug overdose, were over 18 years old, and had been exposed within the previous 24 hours were included. Those aged under 18 years, pregnant women, and those suffering caustic ingestion or heavy metal poisoning were excluded.

The subjects' age, sex, clinical parameters such as vital signs, Glasgow Coma Scale (GCS), underlying diseases, laboratory results, and clinical outcomes were collected. Aspiration pneumonia was defined as newly developed lung lesions on chest X-ray or computed tomography and worsening of respiratory symptoms within 48 hours of admission [9, 10].

This study was approved by the Institutional Review Board (IRB) of the study hospital (IRB no. 20170626/30-2017-15/073). Informed consent was waived by the IRB.

### 2.2. Statistical Analysis

Mortality, endotracheal intubation and vasopressor use, development of aspiration pneumonia, intensive care unit (ICU) admission, and hospital admission were evaluated as clinical outcomes.

A subgroup analysis was performed for factors that can affect the clinical efficacy of activated charcoal based on previous studies [11–13]. Moreover, we defined the conditions under which activated charcoal can be beneficial: (1) patient presents within 2 hours of ingestion, (2) GCS 13-15 on arrival, and (3) potentially toxic ingestion (excluding ingestion of less toxic substance such as benzodiazepines and sedatives, as well as cases of ingestion of relatively small amounts) [5]. Two board-certified emergency physicians decide whether there is potential toxic exposure or not.

The Shapiro-Wilk test was used to evaluate the normality of the continuous variables, which were expressed as a mean ± standard deviation or median (interquartile range), as appropriate. Categorical variables were summarized by frequency according to the corresponding percentage and compared using the chi-square test or Fisher's exact test as appropriate.

All of the analyses were performed with SPSS 22 (IBM, Armonk, New York, USA). A p value less than 0.05 was considered to indicate statistical significance.

## 3. Results

### 3.1. Characteristics of the Patients

In this retrospective cohort study, we identified 634 patients who met the study criteria. Four hundred and thirteen (413) patients were managed during the activated charcoal-available period and 221 during the activated charcoal-unavailable period (Table 1). Activated charcoal was used in the treatment of 141 patients (34.0%) during the activated charcoal-available period. No enrolled patient received multiple-dose charcoal.

There was no interperiod difference in patient age, sex, medical history, or vital signs. There were statistical interperiod differences in some initial laboratory values (sodium, creatine kinase-MB, troponin I, and activated partial thromboplastin time) (Table 1), though they were minimal and not clinically significant. Gastric lavage was more frequently performed in the activated charcoal-available period (26.4%) than in the activated charcoal-unavailable period (10%) (*p* < 0.001). The ingested toxic substances of both groups are presented in Table 2.

### 3.2. Charcoal Availability and Clinical Outcome

There were no differences in the incidence of aspiration pneumonia, the rate of intubation, vasopressor use, or mortality between the charcoal-available and charcoal-unavailable periods (Table 3).

**Figure 1:**
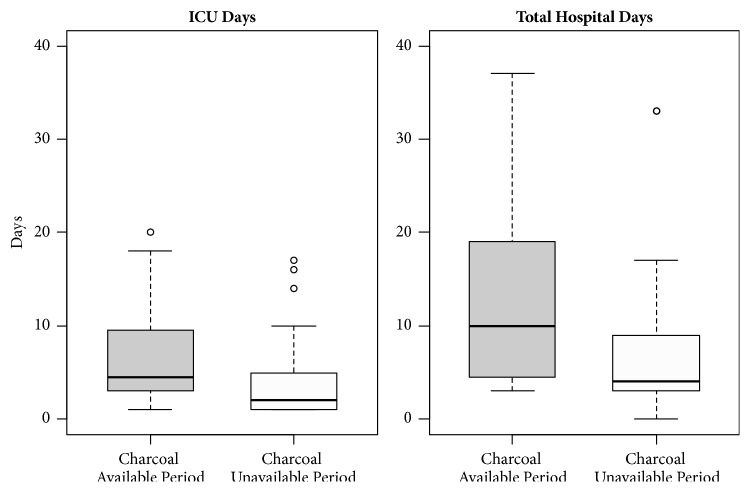
Length of stay of patients admitted to ICU between charcoal-available and charcoal-unavailable periods.^*∗*^   ^*∗*^One extreme outlier case is present in charcoal-unavailable period (41 ICU days and 91 total hospital days).

The rates of hospital admission (43.3 versus 29.9%,* p* < 0.001) and ICU admission (5.8 versus 13.6%,* p* < 0.001) were higher in the charcoal-unavailable period; however, the number of ICU days was lower and the total hospital stay was shorter (Table 3 and Figure 1).

### 3.3. GCS and Clinical Outcomes according to Charcoal Availability

According to the GCS levels, there were no interperiod differences in aspiration pneumonia, intubation, vasopressor use, or mortality. In the charcoal-unavailable period, hospital admission was less common and the rate of ICU admission was higher for patients with preserved mental status (GCS 13-15) (Table 3). During the charcoal-available period, both hospital admission and ICU admission were more common for charcoal-administered patients (Table 4).

### 3.4. Single- and Multiple-Drug Ingestions and Clinical Outcome according to Activated Charcoal Availability

Higher hospital admission rates and lower ICU admission rates during the charcoal-unavailable period were observed among the single-drug-poisoned patients. The other clinical outcomes did not differ between the periods for either single- or multiple-drugs-poisoned patients (Table 5).

### 3.5. Presenting Time and Clinical Outcome according to Activated Charcoal Availability

Higher hospital admission rates and lower ICU admission rates during the charcoal-unavailable period also were observed among the patients with a time delay of more than 1 hour from ingestion to ED visit (Table 6). The other clinical outcomes did not differ between the periods. During the charcoal-available period, intubations were more commonly conducted for patients who had arrived at the ED within 1 hour and received activated charcoal (11.4 versus 9.1%,* p* = 0.015) (Table 6).

### 3.6. Clinical Outcomes of Patients Who May Benefit from Activated Charcoal

Twenty-three patients and 17 patients were identified during the charcoal-available and charcoal-unavailable periods, respectively. Activated charcoal was used for 12 patients (52.1%) during the charcoal-available period. There were no differences in clinical outcomes between the periods (Table 7) (Supplemental Table 2).

### 3.7. Mortality Cases

Among the mortality cases, only one patient visited within 2 hours of exposure. His age was 92 and he died due to aspiration pneumonia. Charcoal was not used, because of decreased consciousness, sedative poisoning, and high risk of respiratory complication. He died from respiratory complications 18 days from ED visit (Table 8). Other patients visited the ED 4 hours or more after exposure to toxins (Table 8).

## 4. Discussion

Activated charcoal has been used for the treatment of poisoned patients for more than 100 years and remains the major GI decontamination therapy for such cases [1, 2, 5, 14].

Many preclinical studies have shown beneficial effects of activated charcoal in various kinds of drug poisonings [5, 15–19]. However, few clinical studies have established any clinical benefits of activated charcoal use [1, 2, 5, 14]. One retrospective study showed activated charcoal within 2 hours of a paracetamol ingestion is associated with a decreased requirement for N-acetylcysteine [20]. A recent prospective study on massive paracetamol overdose found a benefit of activated charcoal use within 4 hours. Within that time, development of hepatotoxicity (peak ALT > 1000 U/L) was lower in the charcoal-treated patients. However, only serum liver enzyme levels were evaluated as an outcome and mortality, hospital day, presence and severity of hepatic encephalopathy, and liver transplantation were not [21].

Various clinical studies have failed to prove any clinical benefits of activated charcoal [8, 13, 22–24]. In a prospective ED study, there was no improvement of the clinical outcomes of single-dose activated charcoal. Activated charcoal use was associated with longer ED stay and higher incidence of vomiting. However, ICU admission, length of ICU and hospital stay, length of intubation time, and development of aspiration pneumonia were found to be unrelated to activated charcoal use [13]. One recent prospective study showed that age was the only factor associated with clinical improvement in case of drug poisoning, activated charcoal administration was determined to be unrelated to clinical outcome [24].

The reasons for the discrepancies between the results of preclinical and clinical studies are not clear. The risk of charcoal-induced aspiration might be one explanation. Chemical pneumonitis due to direct charcoal exposure is a fatal complication and has been, to say the least, a major concern [5, 25–29]. Development of aspiration pneumonitis in overdose patients has been related to poor prognosis [10, 11]. Activated charcoal use in instance of nonintubation and decreased mental status has been related to aspiration pneumonia [10]. However, other clinical studies have shown minimal risk of aspiration pneumonitis and pneumonia in the use of activated charcoal [30–32]. One retrospective study found that the incidence of pulmonary aspiration was only 0.6% in patients who had received multiple doses of activated charcoal [30]. An analysis of a toxicology-unit admission cohort showed a prevalence of aspiration pneumonitis of 11% and established that the predictors did not include activated charcoal use but rather age, emesis, and time delay from ingestion to hospital [32].

It is well known that airway protection is important for prevention of aspiration pneumonitis in poisoned patients [9]. The current guidelines emphasize airway protection prior to activated charcoal use [1, 2, 5]. In our study, intubations also were conducted more frequently for charcoal-administered patients (18, 51.4%) than for not-administered patients (17, 6.3%) (*p *= 0.024) in the charcoal-available period.

In our study, mortality, need of vasopressor, intubation, and incidence of aspiration pneumonia were not affected by charcoal availability (Table 3). Within the charcoal-available period, aspiration pneumonia developed in 13 (9.2%) of the charcoal-administered patients and 28 (10.3%) of the not-administered patients, of which difference was not significant (*p* = 0.729).

The ICU admission rate was increased in the charcoal-unavailable periods. However, the total hospital admission rate was lower (Table 3). For patients admitted to ICU, the number of ICU days and total hospital days were shorter in the charcoal-unavailable period (5 [3–10] versus 2 [1–5],* p* = 0.010 and, 10 [4–19] versus 4 [3–9],* p* = 0.021, respectively) (Figure 1). Because ICU days were shorter in the charcoal-unavailable period, the increase in the ICU admission rate might have been the result of concerns about activated charcoal unavailability.

In our study, most of the deaths occurred when the visit to the ED was delayed (Table 8). Delayed ED presentation can cause worsening of poisoning [33]. Early adequate supportive care seems to be a more cardinal treatment process than activated charcoal use.

The rate of activated charcoal use was high in charcoal-available period (34.1%). Nearly half (42%) of patients received the activated charcoal more than 2 hours after exposure to toxins. A Norwegian study reported 16% activated charcoal use for all admitted acute-poisoning patients in Oslo [34, 35]. However, our study also showed a low mortality rate in both the charcoal-available and charcoal-unavailable periods. These findings indirectly show that adequate supportive care is essential to the treatment of oral drug poisoned patients.

There are several limitations to this study. First, it is a retrospective study. Neither randomization or nor blinding was applied, and clinical decisions might have been affected by charcoal-availability. Second, the severity of poisoning was not high; the total mortality was only 1.6% (10 patients). Considering that activated charcoal is more beneficial for severe poisoning, its effect might have been underestimated [1, 2]. High mortality was observed among the delayed ED visit patients. Third, we included only adult patients and oral drug poisonings other than from plants, mushrooms, herbicides, and pesticides. Fourth, gastric lavage was used as a GI decontaminating agent in both periods and which can attenuate the clinical effects of charcoal unavailability. Finally, the exact substances or amounts could not be identified in many cases and thus were not fully evaluated in this study.

## 5. Conclusions

Between the charcoal-available and charcoal-unavailable periods, activated charcoal availability was unrelated to mortality, incidence of aspiration pneumonia, intubation, or use of vasopressor for treatment of oral drug poisoned patients.

## Figures and Tables

**Table 1:** Patient characteristics.

	Charcoal-available period	Charcoal-unavailable period	*p*-value
	(n=413)	(n=221)	
Age, median (IQR)	41 (28-58)	43	0.267
Male, n (%)	148 (35.8%)	77	0.803
Medical History			
Hypertension, n (%)	72 (17.4%)	32 (14.5%)	0.339
Diabetes mellitus, n (%)	40 (9.7%)	19 (8.6%)	0.653
Multiple drug ingestion	242 (58.6%)	119 (53.8%)	0.250
Vital signs			
Mean blood pressure (mmHg), median (IQR)	93 (79-103)	94 (77-107)	0.495
Heart rate (/min), median (IQR)	90 (79-100)	87 (76-103)	0.258
Respiratory rate (/min), median (IQR)	20 (18-20)	20 (18-20)	0.168
Body temperature (°C), median (IQR)	36.2 (36.0-36.6)	36.4 (36.0-36.7)	0.102
GCS, median (IQR)	14 (11-15)	14 (10-15)	0.971
Laboratory results			
WBC (x10^3^/*μℓ*), median (IQR)	7.55 (5.76-10.19)	7.44 (5.96-9.82)	0.996
Hemoglobin (g/d*ℓ*), median (IQR)	13.5 (12.6-14.6)	13.4 (12.2-14.6)	0.374
Platelet (*∗*1000/*μℓ*), median (IQR)	249 (205.5-299.5)	249 (202-298.5)	0.970
Sodium (mmol/*ℓ*), median (IQR)	139.1 (137.3-140.9)	138.6 (136.6-140.6)	0.046
Potassium (mmol/*ℓ*), median (IQR)	3.8 (3.54-4.12)	3.8 (3.50-4.00)	0.429
Total CO_2_ (mmol/*ℓ*), median (IQR)	22.8 (20.90-26.26)	22.3 (20.15-26.50)	0.082
BUN (mg/d*ℓ*), median (IQR)	12 (9-16)	13 (11-16)	0.167
Creatinine (mg/d*ℓ*), median, (IQR)	0.74 (0.61-0.89)	0.74 (0.65-0.91)	0.242
AST (IU/*ℓ*), median, (IQR)	25 (19-37)	25 (19-28)	0.880
ALT (IU/*ℓ*), median, (IQR)	15 (10-25)	15 (10-29)	0.772
CRP (mg/d*ℓ*), median, (IQR)	0.08 (0.03-0.24)	0.07 (0.02-0.26)	0.501
CK (IU/*ℓ*), median, (IQR)	97 (69-151)	96 (68-159)	0.838
CK-MB (ng/mL), median (IQR)	0.5 (0.5-1.1)	0.9 (0.6-2.1)	< 0.001
Troponin I (ng/mL), median (IQR)	0.04 (0.04-0.04)	0.02 (0.01-0.02)	< 0.001
aPTT (seconds), median (IQR)	25.9 (23.0-28.7)	27.2 (25.0-30.3)	< 0.001
PT (INR), median (IQR)	1.06 (1.01-1.11)	1.06 (1.01-1.12)	0.836
Gastric lavage, n (%)	109 (26.4%)	22 (10%)	< 0.001
Activated charcoal, use n (%)	141 (34.1%)	0 (0%)	< 0.001
SOFA score*∗*	6 (4-7)	5 (3-6)	0.153

GCS: Glasgow Coma Scale; WBC: white blood cells; BUN: blood urea nitrogen; AST: aspartate transaminase; ALT: alanine aminotransferase; CRP: C-reactive protein; CK: creatine kinase; CK-MB: creatine kinase-MB; aPTT: activated partial thromboplastin time; PT: prothrombin time; SOFA: sequential organ failure assessment.

*∗*Only for ICU patients.

**Table 2:** Toxic substances ingested by patients.

	Charcoal-available period	Charcoal-unavailable period
CNS Affecting Drug, n (%) *∗*	134 (32.4%)	77 (34.8%)
Benzodiazepine, n (%)	21 (5.1%)	8 (3.6%)
Acetaminophen, n (%)	16 (3.9%)	8 (3.6%)
Cardiovascular drug, n (%)	4 (1.0%)	4 (1.8%)
Salicylate, n (%)	6 (1.5%)	1 (0.5%)
OTC drugs, n (%) †	9 (2.2%)	7 (3.2%)
Two or more toxin types, n (%)	132 (32.0%)	73 (33.0%)
Others, n (%)	51 (12.3%)	26 (11.8%)
Unknown, n (%)	40 (9.7%)	17 (7.7%)

CNS: central nervous system; OTC: over the counter.

*∗*Except benzodiazepine.

^†^Substances not clearly identified.

**Table 3:** Charcoal availability and clinical outcome.

	Charcoal-available period	Charcoal-unavailable period	*p*-value
Aspiration pneumonia, n (%)	41 (9.9%)	21 (9.5%)	0.864
Endotracheal intubation, n (%)	35 (8.5%)	16 (7.2%)	0.586
Vasopressor, n (%)	22 (5.3%)	11 (5.0%)	0.850
Mortality, n (%)	8 (1.9%)	2 (0.9%)	0.507
Hospital admission, n (%)	179 (43.3%)	66 (29.9%)	<0.001
ICU admission, n (%)	24 (5.8%)	30 (13.6%)	<0.001

ICU: intensive care unit.

**Table 4:** GCS and clinical outcomes compared between charcoal-available and charcoal-unavailable periods.

	Charcoal-available period				Charcoal-unavailable period	*p-*value^†^
	Non-charcoal	Charcoal	Total	*p-*value*∗*		
Aspiration pneumonia						
GCS 13-15	7 (4.0%)	7 (7.4%)	14 (5.2%)	0.258	3 (2.1%)	0.119
GCS 9-12	7 (13.7%)	3 (10.7%)	10 (12.7%)	>0.990	4 (10.8%)	>0.999
GCS 3-8	14 (29.2%)	3 (15.8%)	17 (25.4%)	0.356	14 (36.8%)	0.216
Intubation						
GCS 13-15	3 (1.7%)	6 (6.4%)	9 (3.4%)	0.071	3 (2.1%)	0.552
GCS 9-12	5 (9.8%)	4 (14.3%)	9 (11.4%)	0.713	3 (8.1%)	0.749
GCS 3-8	9 (18.8%)	8 (47.1%)	17 (25.4%)	0.064	10 (26.3%)	0.915
Vasopressor use						
GCS 13-15	7 (4.0%)	3 (3.2%)	10 (3.7%)	>0.990	5 (3.4%)	0.868
GCS 9-12	4 (7.8%)	1 (3.6%)	5 (6.3%)	0.651	1 (2.7%)	0.663
GCS 3-8	6 (12.5%)	1 (5.3%)	7 (10.4%)	0.663	5 (13.2%)	0.753
Mortality						
GCS 13-15	3 (1.7%)	1 (1.1%)	4 (1.5%)	>0.990	1 (0.7%)	0.660
GCS 9-12	1 (2.0%)	0 (0%)	1 (1.3%)	>0.990	1 (2.7%)	0.538
GCS 3-8	3 (6.3%)	0 (0%)	3 (4.5%)	0.553	0 (0%)	0.552
Hospital admission						
GCS 13-15	50 (28.9%)	39 (41.5%)	89 (33.3%)	0.037	28 (19.2%)	0.002
GCS 9-12	26 (51.0%)	17 (60.7%)	43 (54.4%)	0.046	15 (40.5%)	0.163
GCS 3-8	36 (75.6%)	11 (57.9%)	47 (70.1%)	0.168	23 (60.5%)	0.315
ICU admission						
GCS 13-15	2 (1.2%)	6 (6.4%)	8 (3.0%)	0.024	12 (8.2%)	0.028
GCS 9-12	3 (5.9%)	2 (7.1%)	5 (6.3%)	>0.990	6 (16.2%)	0.102
GCS 3-8	7 (14.6%)	4 (21.1%)	11 (16.4%)	0.492	12 (31.6%)	0.071
Number of patients						
GCS 13-15	173	94	267		146	
GCS 9-12	51	28	79		37	
GCS 3-8	48	19	67		38	

GCS: Glasgow Coma Scale; ICU: intensive care unit.

^*∗*^between noncharcoal and charcoal.

^†^Between charcoal-available and charcoal-unavailable periods.

**Table 5:** Multiple-drug ingestion and clinical outcomes compared between charcoal-available and charcoal-unavailable periods.

	Charcoal-available period				Charcoal -unavailable period	*p-*value^†^
	Non-Charcoal	Charcoal	Total	P value^*∗*^		
Aspiration pneumonia						
single drug	11 (9.9%)	8 (13.3%)	19 (11.1%)	0.497	11 (10.8%)	0.933
multiple drug	17 (10.6%)	5 (6.2%)	22 (9.1%)	0.263	10 (8.4%)	0.829
Intubation						
single drug	8 (7.2%)	9 (15.0%)	17 (9.9%)	0.104	8 (7.8%)	0.561
multiple drug	9 (5.6%)	9 (11.1%)	18 (7.4%)	0.122	8 (6.7%)	0.805
Vasopressor use						
single drug	5 (4.5%)	1 (1.7%)	6 (3.5%)	0.666	7 (6.9%)	0.245
multiple drug	12 (7.5%)	4 (4.9%)	16 (6.6%)	0.588	4 (3.4%)	0.204
Mortality						
single drug	3 (2.7%)	0 (0%)	3 (1.8%)	0.553	1 (1.0%)	>0.999
multiple drug	4 (2.5%)	1 (1.2%)	5 (2.1%)	0.667	1 (0.8%)	0.668
Hospital admission						
single drug	49 (44.1%)	30 (50.0%)	79 (46.2%)	0.464	33 (32.4%)	0.024
multiple drug	63 (39.1%)	37 (45.7%)	100 (41.3%)	0.329	33 (24.8%)	0.012
ICU admission						
single drug	2 (1.8%)	7 (11.7%)	9 (5.3%)	0.010	16 (15.7%)	0.004
multiple drug	10 (6.2%)	5 (6.2%)	15 (6.2%)	0.991	14 (11.8%)	0.067
Number of patients						
single drug	111	60	171		102	
multiple drug	161	81	242		119	

GCS: Glasgow Coma Scale; ICU: intensive care unit.

^*∗*^between noncharcoal and charcoal.

^†^Between charcoal-available and charcoal-unavailable period.

**Table 6:** Time delay from drug ingestion to ED visit and clinical outcome between charcoal-available and charcoal-unavailable periods.

	Charcoal-available period				Charcoal-unavailable period	*p-*value^†^
	Non-charcoal	Charcoal	Total	*p*value^*∗*^		
Aspiration pneumonia						
within 1hr	5 (7.6%)	3 (6.8%)	8 (7.3%)	1	5 (7.8%)	0.818
over 1hr	23 (11.2%)	10 (10.3%)	33 (10.9%)	0.823	16 (10.2%)	>0.999
Intubation						
within 1hr	6 (9.1%)	5 (11.4%)	11 (10%)	0.015	3 (4.7%)	0.214
over 1hr	11 (5.3%)	13 (13.4%)	24 (7.9%)	0.697	13 (8.3%)	0.893
Vasopressor use						
within 1hr	1 (1.5%)	0 (0%)	1 (0.9%)	1	3 (4.7%)	0.141
over 1hr	16 (7.8%)	5 (5.2%)	21 (6.9%)	0.404	8 (5.1%)	0.443
Mortality						
within 1hr	0 (0%)	0 (0%)	0 (0%)	NA	0 (0%)	N/A
over 1hr	7 (3.4%)	1 (1.0%)	8 (2.6%)	0.231	2 (1.3%)	0.506
Hospital admission						
within 1hr	17 (25.8%)	20 (45.5%)	37 (33.6%)	0.339	15 (23.4%)	0.156
over 1hr	95 (46.1%)	47 (48.5%)	142 (46.9%)	0.171	51 (32.5%)	0.003
ICU admission						
within 1hr	3 (4.5%)	4 (9.1%)	7 (6.4%)	0.434	7 (10.9%)	0.285
over 1hr	9 (4.4%)	8 (8.2%)	17 (5.6%)	0.171	23 (14.6%)	0.001
Number of patients						
within 1hr	66	44	110		64	
over 1hr	206	97	303		157	

GCS: Glasgow Coma Scale; ICU: intensive care unit.

^*∗*^between noncharcoal and charcoal.

^†^Between charcoal-available and charcoal-unavailable period.

**Table 7:** Clinical outcomes of patients who may benefit from activated charcoal use*∗*.

	Charcoal-available period	Charcoal-unavailable period	*p*-value
	n=23	n=17	
Aspiration pneumonia, n (%)	2 (8.7%)	1 (5.9%)	0.615
Endotracheal intubation, n (%)	1 (4.3%)	1 (5.9%)	>0.999
Endotracheal intubation after 4 hours from ED visit, n (%)	1 (4.3%)	0 (0%)	>0.999
Vasopressor, n (%)	0 (0%)	2 (11.8%)	0.174
Vasopressor after 4 hours from ED visit, n (%)	0 (0%)	1 (5.9%)	0.436
Mortality, n (%)	0 (0%)	0 (0%)	NA
Hospital admission, n (%)	8 (34.8%)	5 (29.4%)	0.072
ICU admission, n (%)	1 (4.3%)	3 (17.6%)	0.294
Prolonged ICU admission	1 (4.3%)	1 (5.9%)	>0.999

ICU: intensive care unit.

*∗*: (1) present within 2 hours of acute overdose, (2) GCS 13-15 on arrival, and (3) potential toxic ingestion.

**Table 8:** Summary of mortality cases.

Period	Sex	Age	Activated charcoal use	Time delay	Survival time	Toxic substances
Charcoal-available	male	92	-	2 hours	18 days	Sedatives
Charcoal-available	female	78	-	4 hours	2 days	Unidentified
Charcoal-available	male	29	-	17 hours	16 hours	Salicylate
Charcoal-available	female	66	-	8 hours	12 hours	Antidepressants
Charcoal-available	female	17	-	7 hours	7 hours	Bupropion
Charcoal-available	male	72	-	12 hours	1 days	Antipsychotics
Charcoal-available	female	70	-	6 hours	12 hours	Multiple drug including betablocker
Charcoal-available	male	56	+	4 hours	10 days	Multiple unidentified drugs including sedatives
Charcoal-unavailable	male	77	-	6 hours	4 days	Multiple antipsychotics
Charcoal-unavailable	male	61	-	4 hours	3 days	Unknown

## Data Availability

The data used to support the findings of this study are available from the corresponding author upon request.

## Supplementary Materials

Supplementary Materials Supplemental Table 1: time delay from drug exposure to presentation. Supplemental Table 2: major toxic agents of patients who may benefit from activated charcoal*∗*. NSAID: Nonsteroidal Anti-Inflammatory Drugs. *∗*: (1) present within 2 hours of acute overdose, (2) GCS 13-15 on arrival, and (3) potential toxic ingestion.
