# Supplementary material for: Clinical Effects of Activated Charcoal Unavailability on Treatment Outcomes for Oral Drug Poisoned Patients
Source: Emerg Med Int. 2018 Oct 3;2018:4642127. doi: 10.1155/2018/4642127 (PMC6192078; doi:10.1155/2018/4642127)
Supplement: Supplementary Materials — Supplemental Table 1: time delay from drug exposure to presentation. Supplemental Table 2: major toxic agents of patients who may benefit from activated charcoal∗. NSAID: Nonsteroidal Anti-Inflammatory Drugs. ∗: (1) present within 2 hours of acute overdose, (2) GCS 13-15 on arrival, and (3) potential toxic ingestion. [file 4642127.f1.docx]

Supplemental Table 1.

Time delay from drug exposure to presentation

|  |  | ≤1hr | ≤2hr | ≤4hr | ≤6hr | ≤12h | ≤24hr |
| --- | --- | --- | --- | --- | --- | --- | --- |
| Pre | Total | 124 (30%) | 56 (14%) | 57 (14%) | 51 (12%) | 61 (15%) | 64 (15%) |
|  | Charcoal (+) | 51 (36%) | 29 (21%) | 28 (20%) | 18 (13%) | 12 (9%) | 3 (2%) |
| Post |  | 63 | 41 | 34 | 15 | 27 | 23 |

Supplemental Table 2.

Major toxic agents of patients who may benefit from activated charcoal*.

| Toxic agent | n (%) |
| --- | --- |
| Antiepileptics | 5 (12.5%) |
| Tricyclic antidepressants | 2 (5%) |
| Antipsychotics | 12 (30%) |
| Antihypertensives | 4 (10%) |
| NSAIDs | 4 (10%) |
| Acetaminophen | 3 (7.5%) |
| Oral Hypoglycemic Agents | 1 (2.5%) |
| Unidentified | 9 (22.5%) |
| Total | 40 |

NSAID: Nonsteroidal Anti-inflammatory Drugs

* 1) present within 2 hours of acute overdose, 2) GCS 13-15 on arrival, and 3) potential toxic ingestion
